# Comparative effectiveness of ertapenem versus other empirical antibiotics in elderly patients with complicated intra-abdominal infection: a real-world inverse probability of treatment weighting study

**DOI:** 10.3389/fcimb.2025.1759636

**Published:** 2026-01-16

**Authors:** Yao Sun, Yiming Guo, Haizhen Cui, Yueyao Jiang, Qian Yu

**Affiliations:** 1 Department of Pharmacy, China–Japan Union Hospital of Jilin University, Changchun, China; 2 School of Pharmaceutical Sciences, Jilin University, Changchun, China

**Keywords:** antimicrobial stewardship, comparative effectiveness, complicated intra-abdominal infection, elderly patients, empirical antibiotic therapy, ertapenem, inverse probability of treatment weighting, real-world study

## Abstract

**Background:**

Elderly adults with complicated intra-abdominal infection (cIAI) represent a functionally immunocompromised population due to immunosenescence, multimorbidity, and frailty. Optimizing empirical antibiotic therapy in this group is essential to improve outcomes while minimizing unnecessary broad-spectrum antimicrobial exposure and antimicrobial resistance (AMR) selection pressure. Ertapenem is a once-daily carbapenem with favorable pharmacological properties, yet contemporary real-world comparative data in older adults are limited.

**Methods:**

We conducted a retrospective, real-world comparative-effectiveness study of hospitalized adults aged ≥65 years with cIAI at a tertiary academic medical center from 2019 to 2025. Eligible patients received empirical monotherapy with ertapenem, meropenem, cefoperazone–sulbactam, or moxifloxacin for ≥72 hours. A multinomial propensity score-based inverse probability of treatment weighting (IPTW) approach was used to balance baseline covariates across the four regimens. The primary outcome was clinical cure or improvement. Secondary outcomes included all-cause in-hospital mortality, infection-related mortality, intra-abdominal infection–related mortality, duration of antibiotic treatment, and length of hospitalization. Prespecified subgroup analyses were conducted by age group (65–70, 71–80, ≥81 years) and infection source (gastrointestinal *vs* non-gastrointestinal).

**Results:**

A total of 609 patients met eligibility criteria: 129 received ertapenem, 135 meropenem, 125 cefoperazone–sulbactam, and 220 moxifloxacin. IPTW achieved excellent covariate balance, with all standardized mean differences <0.10. In IPTW-adjusted analyses, clinical cure or improvement did not differ significantly between ertapenem and comparator regimens, with adjusted risk differences ranging from 1.80% to 6.44% (all 95% confidence intervals including zero). Mortality outcomes were likewise comparable across groups. Subgroup analyses suggested that ertapenem was associated with higher cure rates and lower mortality in patients aged 65–70 years and those with non-gastrointestinal infection sources, although confidence intervals were wide, and these findings should be interpreted as exploratory. Differences in secondary outcomes varied across regimens.

**Conclusion:**

In this IPTW-adjusted real-world analysis of elderly adults with cIAI, ertapenem demonstrated clinical effectiveness comparable to meropenem, cefoperazone–sulbactam, and moxifloxacin. Given its once-daily dosing convenience and narrower ecological impact, ertapenem may represent a reasonable and stewardship-aligned empirical option for selected older patients. Prospective and multicenter studies incorporating microbiological and illness severity data are needed to validate these findings.

## 1 Introduction

Complicated intra-abdominal infections (cIAIs) remain a major cause of morbidity and mortality worldwide and require timely source control and appropriate empirical antimicrobial therapy. International guidelines from the Surgical Infection Society (SIS), Infectious Diseases Society of America (IDSA), and World Society of Emergency Surgery (WSES) emphasize the importance of selecting effective empiric antibiotics based on likely pathogens, illness severity, and local resistance epidemiology (Solomkin et al., 2010; Mazuski et al., 2017; Sartelli et al., 2017a, 2021). Multinational cohort studies further highlight the global burden, clinical heterogeneity, and persistently high mortality associated with cIAI, underscoring the need for early and appropriate antimicrobial treatment (Sartelli et al., 2014; Silva-Nunes and Cardoso, 2019; Sartelli et al., 2024).

Elderly adults represent a particularly vulnerable population in this context. Age-related physiological changes, including diminished organ reserve, altered pharmacokinetics, multimorbidity, and frailty, complicate both diagnosis and treatment and are associated with poorer clinical outcomes (Clegg et al., 2013; Arvaniti et al., 2022). Immunosenescence, the progressive deterioration of innate and adaptive immune function with aging, further increases susceptibility to severe infections, sepsis, and mortality (Crooke et al., 2019). Consequently, empirical antibiotic selection in elderly patients must balance the need for adequate early coverage with the potential risks associated with unnecessary broad-spectrum antimicrobial exposure, adverse events, and antimicrobial resistance (AMR) selection pressure (Kurup et al., 2014; Sartelli et al., 2014, 2016).

Ertapenem, meropenem, cefoperazone–sulbactam, and moxifloxacin are widely used empirical monotherapy options for complicated cIAI, as recommended across international guidelines and regional expert consensus statements (Solomkin et al., 2010; Sartelli et al., 2017a). These agents differ considerably in antimicrobial spectrum, ecological impact, and antimicrobial stewardship considerations, particularly regarding selection pressure for resistant Gram-negative pathogens (Solomkin et al., 2010; Sartelli et al., 2016). Randomized controlled trials and meta-analyses in mixed-age adult populations have demonstrated that ertapenem achieves clinical outcomes comparable to broader-spectrum agents such as piperacillin–tazobactam for community-acquired cIAI (Solomkin et al., 2003; Namias et al., 2007; Falagas et al., 2008). However, most of these pivotal studies were conducted more than a decade ago, before contemporary AMR patterns emerged, and they rarely included sufficient numbers of elderly or frail adults to permit age-specific evaluation. As highlighted by recent guideline updates, contemporary real-world comparative-effectiveness analyses remain essential to inform empirical antibiotic selection in older adults, particularly studies designed to address confounding by indication and other biases inherent in observational data (Robins et al., 2000; Austin, 2011).

To address this gap, we conducted a retrospective inverse probability of treatment weighting (IPTW) study comparing the empirical effectiveness of ertapenem, meropenem, cefoperazone–sulbactam, and moxifloxacin in elderly adults hospitalized with cIAI. This study aims to provide contemporary, clinically relevant evidence to support stewardship-aligned empirical therapy in this immunologically vulnerable population.

## 2 Methods

### 2.1 Study design and data source

This retrospective, observational, real-world comparative-effectiveness study was conducted at the China–Japan Union Hospital of Jilin University, a tertiary academic medical center with a high-volume elderly surgical and medical patient population. The study period spanned June 1, 2019, to May 31, 2025, during which all hospitalized adults aged ≥65 years with a diagnosis of cIAI were screened for eligibility.

Patient-level data were obtained from the hospital’s Electronic Medical Record (EMR) and Health Information System (HIS). Extracted variables included demographic information, ICD-10 diagnostic codes, surgical and procedural records, vital signs, radiological findings, laboratory parameters, antimicrobial administration records (drug name, dosage, frequency, timing, and duration), clinical course documentation, and discharge outcomes. To ensure standardized data abstraction, a structured case report form (CRF) was developed *a priori*, incorporating predefined rules for exposure classification, covariate definitions, and outcome adjudication. Missing data were assessed before analysis, and patients lacking essential baseline covariates required for IPTW were excluded according to predefined criteria.

The primary causal estimand of this study was the average treatment effect (ATE), defined as the expected difference in outcomes if the entire study population of elderly patients with complicated intra-abdominal infection were treated with ertapenem compared with each alternative empirical regimen. Accordingly, the study was designed to estimate population-average comparative effectiveness rather than effects within specific treated subgroups.

All data were fully anonymized before analysis. The study was approved by the Ethics Committee of the China–Japan Union Hospital of Jilin University (Approval No. 2025111802). Owing to the retrospective design and exclusive use of de-identified clinical information, the requirement for informed consent was waived.

### 2.2 Patient selection and group assignment

All hospitalized adults aged ≥65 years during the study period with a diagnosis of cIAI were screened for eligibility. cIAI was identified through a combination of ICD-10 diagnostic codes, radiological findings, operative notes, and physician documentation. To ensure diagnostic accuracy, patients were required to have clinical evidence of intra-abdominal infection extending beyond a single organ along with supportive imaging or intraoperative confirmation, consistent with international guideline definitions (Solomkin et al., 2010; Sartelli et al., 2017b).

Patients were included if they received empirical monotherapy with one of four antibiotics within the first 24 hours of admission: ertapenem, meropenem, cefoperazone–sulbactam, or moxifloxacin. The selection of these regimen options aligns with guideline-endorsed empirical therapy recommendations for adults with cIAI (Solomkin et al., 2010; Sartelli et al., 2017b). To ensure adequate therapeutic exposure and avoid misclassification of brief or trial regimens, patients were required to have received the initial empirical regimen for at least 72 hours unless clinical deterioration or documented intolerance mandated early modification (Solomkin et al., 2003; Bai et al., 2024; Liu et al., 2025). The first administered agent formed the basis for treatment group assignment.

Patients who switched empirical antibiotics within the first 72 hours were excluded to ensure adequate exposure to the initially prescribed regimen and to reduce exposure misclassification. In routine clinical practice, response to empirical antimicrobial therapy is typically assessed after an initial treatment window, and very early switching may reflect diagnostic uncertainty or incomplete exposure rather than failure of the empirical strategy (Solomkin et al., 2010; Mazuski et al., 2017). This restriction aligns with an intention-to-treat–type estimand, which focuses on outcomes associated with initiating a given empirical regimen with a minimum exposure period in observational causal analyses (Hernan and Robins, 2020).

Exclusion criteria were established to reduce heterogeneity and ensure data completeness. Patients were excluded if they: (1) were younger than 65 years; (2) lacked radiological or operative evidence supporting the diagnosis of cIAI; (3) received more than one systemic antibiotic concurrently for >72 hours during initial empiric treatment; (4) had missing baseline laboratory or imaging variables required for propensity score modeling; (5) had terminal malignancy, advanced palliative status, or were admitted solely for end-of-life care, as these patients may experience mortality unrelated to infectious disease processes; or (6) lacked documentation for primary or secondary outcomes. Patients hospitalized for <24 hours or transferred with insufficient clinical data were also excluded.

**Figure 1:**
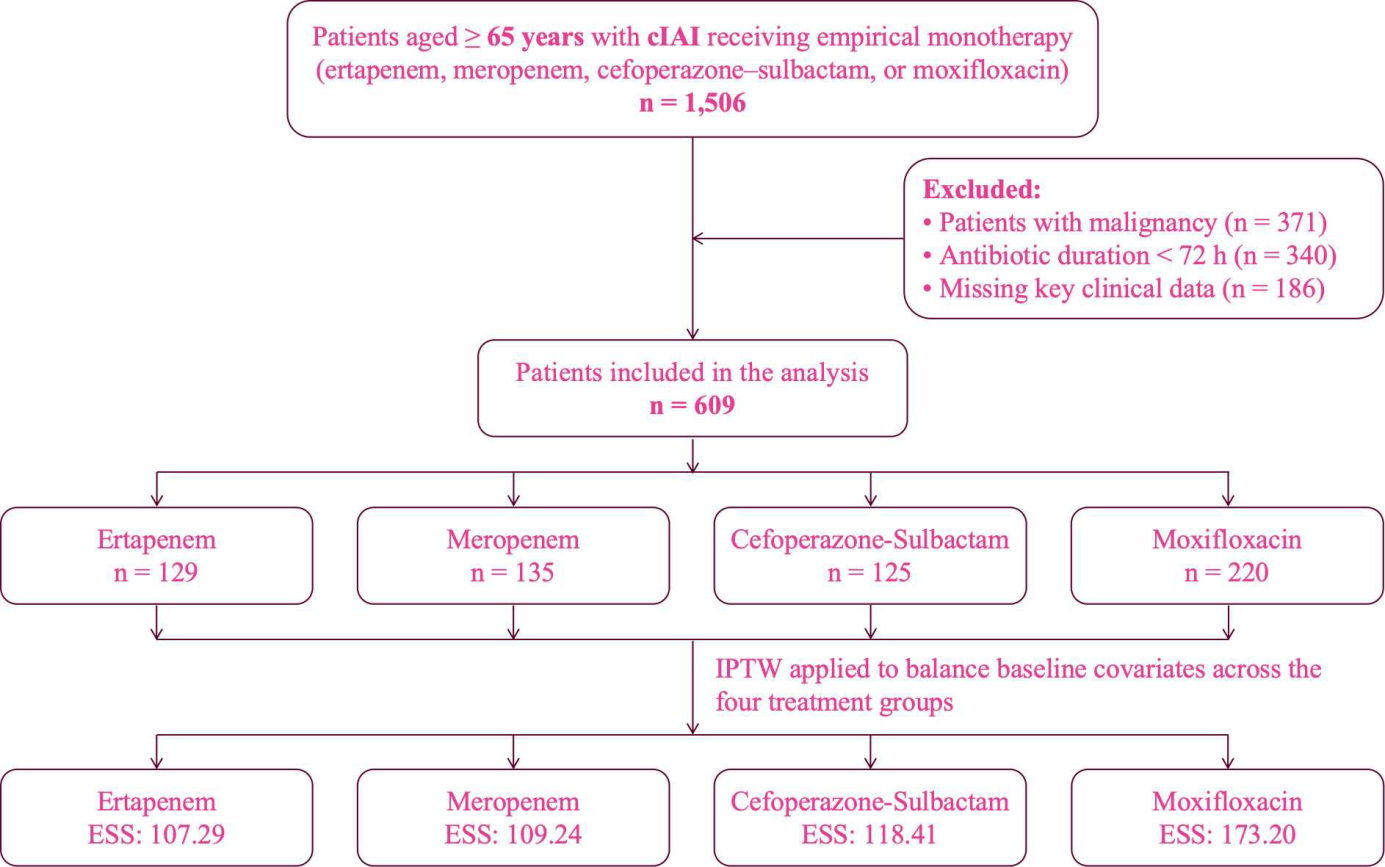
Patient selection flowchart and distribution of treatment groups before and after IPTW. IPTW, inverse probability of treatment weighting; ESS, effective sample size.

Eligible patients were assigned to one of four mutually exclusive treatment groups according to the empirical antibiotic initiated: ertapenem, meropenem, cefoperazone–sulbactam, or moxifloxacin. No patients who switched antimicrobials within the first 72 hours were included, ensuring that treatment assignment reflected the initial empirical clinical decision. A flow diagram summarizing patient identification, exclusions, and final cohort construction is presented in **Figure 1**.

### 2.3 Definitions

cIAI was defined according to internationally accepted criteria as an infection that extends beyond the hollow viscus of origin into the peritoneal space or another otherwise sterile region of the abdominal cavity, often resulting in peritonitis, abscess formation or a localized complex intra-abdominal infection. Diagnosis required both clinical signs (e.g., abdominal pain, fever, peritoneal signs, leukocytosis) and objective evidence from radiological imaging or intraoperative findings (Solomkin et al., 2010; Sartelli et al., 2017b).

#### 2.3.1 Infection source classification

Because the microbial spectrum and clinical course of intra-abdominal infections vary by anatomic origin, we categorized source infections for analysis into gastrointestinal and non-gastrointestinal groups. This classification mirrors the organ-based categories commonly used in large clinical trials and guideline discussions of IAIs (e.g., appendiceal, colonic, biliary, pancreatic) and allows pragmatic grouping for antimicrobial and outcome analyses (Solomkin et al., 2010; Sartelli et al., 2017b). This pragmatic grouping is also consistent with prior observational cIAI studies and reflects the differing microbial profiles between luminal gastrointestinal sources, often dominated by mixed aerobic and anaerobic flora, and hepatobiliary or pancreatic infections, which typically involve distinct pathogen distributions. Gastrointestinal source included infections originating from hollow-viscus structures of the gastrointestinal tract (e.g., stomach/duodenum, small bowel, colon, appendix), perforations, or gastrointestinal–tract–derived abscesses. Non-gastrointestinal source included infections arising from the biliary tract, pancreas, or other solid abdominal organs where the infection does not directly stem from the intestinal lumen.

#### 2.3.2 Treatment exposure

Empirical antimicrobial therapy was defined as the initial antibiotic regimen administered before definitive culture results, consistent with guideline-recommended early management of cIAI. The four regimens under comparison, ertapenem, meropenem, cefoperazone–sulbactam, and moxifloxacin, are recognized as acceptable empiric options depending on clinical presentation, predicted pathogens, and local resistance patterns (Solomkin et al., 2010; Sartelli et al., 2017a).

#### 2.3.3 Primary and secondary outcomes

The primary outcome was clinical cure or improvement during the index hospitalization, defined as a binary endpoint (yes/no). Clinical cure or improvement was considered present if the treating physicians documented resolution or clear improvement of abdominal signs and symptoms, stabilization or normalization of vital signs, and an overall favorable clinical assessment without the need for unplanned escalation of antimicrobial therapy for cIAI. Outcome assessment was based on physician documentation and was independent of treatment assignment. This definition is consistent with outcome measures used in prior randomized and observational cIAI studies (Solomkin et al., 2010; Mazuski et al., 2017).

Secondary outcomes included five clinically relevant endpoints. All-cause in-hospital death was defined as mortality from any cause occurring during the index hospitalization. Infection-related death refers to mortality judged by the treating clinicians as primarily attributable to infectious processes, such as sepsis or septic shock, rather than non-infectious etiologies. A more specific category, intra-abdominal infection–related death, captures fatalities directly resulting from uncontrolled intra-abdominal infectious pathology, including diffuse peritonitis, persistent or recurrent intra-abdominal abscess, or other documented intra-abdominal septic complications.

Two additional secondary outcomes were continuous measures of treatment duration and healthcare utilization. Duration of antibiotic treatment was defined as the total number of hours the initial empirical antimicrobial regimen was administered, measured from the first dose until discontinuation, escalation, or de-escalation. Length of hospitalization was defined as the number of days from admission to discharge during the index inpatient episode. Both variables reflect the clinical course and therapeutic intensity associated with the treatment of elderly patients with cIAI.

### 2.4 Covariates and data collection

Baseline covariates were selected *a priori* based on clinical relevance, guideline recommendations for cIAI management, and their potential influence on empirical antibiotic choice or patient outcomes. All covariates were extracted from the hospital’s EMR and HIS using standardized definitions to ensure consistency.

Demographic variables included age and sex. Clinical presentation variables encompassed the source of cIAI (gastrointestinal *vs* non-gastrointestinal), as documented by treating physicians based on imaging, operative findings, and clinical assessment. The presence of surgical intervention during the index hospitalization, defined as laparoscopic or open operative management for source control, was recorded as a binary variable and used as a baseline covariate rather than an outcome.

Baseline laboratory findings were categorized as normal or abnormal based on hospital reference ranges and included key inflammatory and infection-related markers such as white blood cell count, neutrophil percentage, C-reactive protein, and serum albumin, among others. These laboratory abnormalities were coded as binary indicators (normal *vs* abnormal) for use in the propensity score model. Imaging findings, including CT or ultrasound evidence of abscess, perforation, free fluid, bowel obstruction, or other acute abnormalities, were similarly classified as abnormal *vs* normal according to radiology reports.

All covariates were measured prior to or at the time of initiating empirical antibiotic therapy, ensuring temporal alignment for the propensity score estimation. Missing baseline covariates were assessed before modeling; patients missing essential variables required for propensity score estimation were excluded according to predefined criteria.

These covariates were incorporated into the multinomial propensity score model to balance baseline characteristics across the four empirical treatment groups and reduce confounding by indication in the IPTW-adjusted analyses.

### 2.5 Propensity score estimation and inverse probability of treatment weighting

Because treatment allocation was not randomized, we used a propensity score (PS)–based IPTW approach to reduce confounding and improve comparability across the four empirical antibiotic regimens (ertapenem, meropenem, cefoperazone–sulbactam, and moxifloxacin). The conceptual foundation of the propensity score follows the framework introduced by Rosenbaum and Rubin (Rosenbaum and Rubin, 1983), while the extension to multiple treatment groups is supported by methodological work on multinomial or generalized propensity scores (McCaffrey et al., 2013).

A generalized propensity score was estimated using a multinomial logistic regression model, with treatment group as the dependent variable and clinically relevant baseline covariates, source of cIAI (gastrointestinal *vs* non-gastrointestinal), age, gender, surgical intervention during hospitalization, abnormal imaging findings, and abnormal laboratory markers of systemic inflammation, as independent variables. All covariates were measured before the initiation of empirical therapy to satisfy temporal requirements for valid causal inference (Hernan and Robins, 2020).

For each patient, the model yielded the estimated probability of receiving the actual treatment observed. Stabilized IPTW weights were then constructed to estimate the ATE. The stabilized weight was defined as:


$$SW_{i}=\frac{P(T=T_{i})}{e_{T_{i}}(X_{i})}$$

where P(T=t) denotes the marginal probability of receiving treatment t, and ${\hat{e}}_{i}(t)\;$ is the generalized propensity score for patient i. Stabilized weights help preserve sample size and improve estimator efficiency while reducing the influence of extreme values, as recommended in marginal structural model methodology (Robins et al., 2000; Hernan and Robins, 2020).

### 2.6 Covariate balance assessment

Covariate balance before and after weighting was evaluated using standardized mean differences (SMDs) rather than P-values, because SMDs are sample-size independent and preferred for assessing covariate balance in observational studies (Austin, 2009; 2011). For continuous variables, SMDs were calculated as the difference in (weighted) means divided by the pooled (weighted) standard deviation; for binary variables, SMDs were calculated as the difference in (weighted) proportions divided by the pooled standard deviation.

Because this study involved four treatment groups, pairwise SMDs were computed for all treatment comparisons, and the maximum absolute pairwise SMD for each covariate was used to summarize balance. An absolute SMD <0.1 was prespecified to indicate adequate balance. After confirming that IPTW achieved acceptable balance across all covariates, all subsequent analyses were conducted in the IPTW-weighted pseudo-population using the stabilized weights.

### 2.7 Statistical analysis

Unweighted descriptive statistics were reported for the original cohort, while all causal comparisons were conducted in the IPTW-weighted pseudo-population. The causal contrast of interest was a population-average comparison of initial empirical antibiotic strategies. Specifically, we estimated the expected difference in outcomes if all eligible patients were initiated on ertapenem versus if all were initiated on each comparator regimen. Subsequent treatment escalation or modification was allowed as part of routine clinical care. This contrast reflects an intention-to-treat–type estimand focused on the initial empirical prescribing decision. For the primary outcome and binary secondary outcomes, weighted logistic regression models were used to estimate adjusted odds ratios (ORs) and 95% confidence intervals (CIs). For continuous secondary outcomes, weighted linear regression models estimated adjusted mean differences (MDs) with 95% CIs.

Use of IPTW-weighted regression models and robust (sandwich) variance estimators follows recommended practices in the causal inference literature (Robins et al., 2000; Hernan and Robins, 2020).

All statistical analyses were performed using R software (version 4.1.3; R Foundation for Statistical Computing), and two-sided p-values <0.05 were considered statistically significant, with emphasis placed on effect sizes and confidence intervals rather than hypothesis testing alone.

## 3 Results

### 3.1 Study population and IPTW application

A total of 1,506 hospitalized patients aged ≥ 65 years with a diagnosis of cIAI and receiving empirical monotherapy with ertapenem, meropenem, cefoperazone–sulbactam, or moxifloxacin were initially screened (**Figure 1**). After excluding patients with malignancy (n = 371), antibiotic treatment durations < 72 hours (n = 340), or missing key clinical variables (n = 186), 609 patients were included in the analytical cohort. Among these, 129 received ertapenem, 135 received meropenem, 125 received cefoperazone–sulbactam, and 220 received moxifloxacin.

IPTW was applied to construct a weighted pseudo-population for comparative effectiveness analysis. After weighting, the effective sample sizes (ESS) were 107.29 for ertapenem, 109.24 for meropenem, 118.41 for cefoperazone–sulbactam, and 173.20 for moxifloxacin.

### 3.2 Baseline characteristics before and after weighting

Marked heterogeneity in baseline characteristics was observed across the four empirical treatment groups prior to adjustment (**Table 1**). Differences were most pronounced in the distribution of cIAI source (gastrointestinal *vs*. non-gastrointestinal), the proportion undergoing surgical intervention, and the prevalence of laboratory or imaging abnormalities, with maximum SMDs ranging between 0.12 and 0.69. These imbalances reflect non-random prescribing patterns in routine clinical practice.

**Table 1:** Baseline characteristics of the study population before IPTW.

Characteristic	Ertapenem (n = 129)	Meropenem (n = 135)	Cefoperazone-Sulbactam (n = 125)	Moxifloxacin (n = 220)	Maximum SMD
Age (median, IQR)	73 (68, 79)	73 (70, 78)	72 (68, 79)	71 (68, 77)	0.215
Gender					0.123
Male (%)	79 (61.24)	79 (58.52)	78 (62.40)	124 (56.36)	
Female (%)	50 (38.76)	56 (41.48)	47 (37.60)	96 (43.64)	
Source of cIAI					0.483
Gastrointestinal (%)	99 (76.74)	101 (74.81)	101 (80.80)	203 (92.27)	
Non-gastrointestinal (%)	30 (23.26)	34 (25.19)	24 (19.20)	17 (7.73)	
Surgery (%)	76 (58.91)	59 (43.70)	43 (34.40)	64 (29.09)	0.628
Laboratory abnormalities (%)	96 (74.42)	116 (85.93)	90 (72.00)	124 (56.36)	0.688
Imaging abnormalities (%)	78 (60.47)	91 (67.41)	91 (72.80)	129 (58.64)	0.301

SMD, standardized mean difference; IQR, interquartile range.

**Figure 2:**
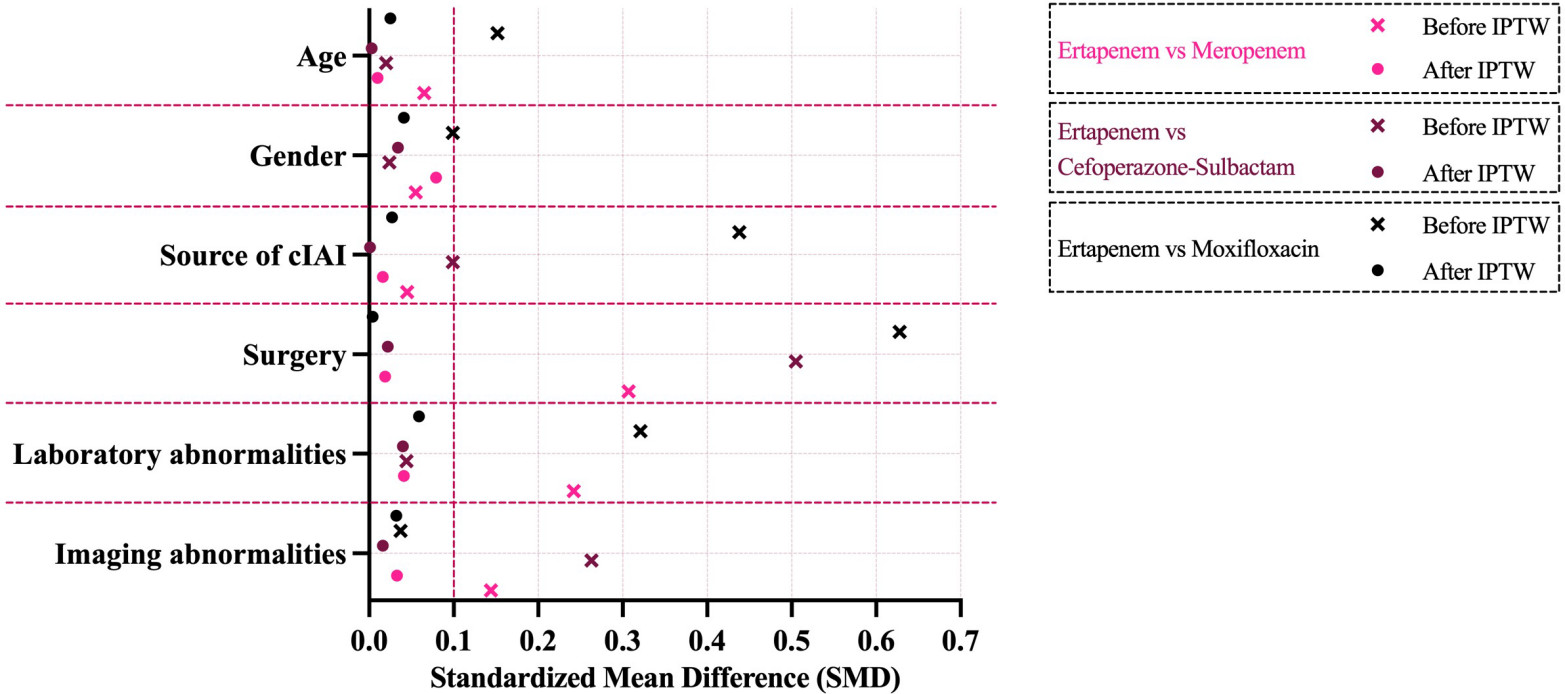
Standardized mean differences for baseline covariates before and after IPTW. IPTW, inverse probability of treatment weighting.

Application of IPTW substantially improved covariate comparability across groups (**Table 2**). Following weighting, all baseline variables demonstrated SMDs < 0.10, indicating excellent balance according to established methodological thresholds. The extent of covariate harmonization achieved through weighting is illustrated in the love plot (**Figure 2**), where post-weighting SMDs are tightly centered around zero with minimal residual dispersion.

**Table 2:** Weighted baseline characteristics after IPTW adjustment.

Characteristic	Ertapenem	Meropenem	Cefoperazone-Sulbactam	Moxifloxacin	Maximum SMD
ESS	107.29	109.24	118.41	173.20	N/A
Age (median, IQR)	73 (68, 79)	73 (70, 77)	72 (68, 79)	72 (68, 78)	0.048
Gender					0.079
Male (%)	58.02	61.89	59.72	60.05	
Female (%)	41.98	38.11	40.28	39.95	
Source of cIAI					0.028
Gastrointestinal (%)	82.22	82.84	82.16	83.22	
Non-gastrointestinal (%)	17.78	17.16	17.84	16.78	
Surgery (%)	40.05	41.01	38.96	39.87	0.042
Laboratory abnormalities (%)	72.09	70.22	70.28	69.43	0.059
Imaging abnormalities (%)	65.18	63.60	64.43	63.66	0.033

SMD, standardized mean difference; ESS, effective sample size; IQR, interquartile range.

### 3.3 Unweighted primary and secondary outcomes

Before IPTW adjustment, notable crude differences in clinical outcomes were observed across the four treatment groups (**Table 3**). The clinical cure or improvement rate was highest in the ertapenem group (96.12%), whereas the meropenem, cefoperazone–sulbactam, and moxifloxacin groups demonstrated cure rates of 86.67%, 90.40%, and 95.00%, respectively. Correspondingly, their unadjusted RDs, calculated as ertapenem minus comparator, were 9.46 (95% CI, 2.83 to 16.09) for meropenem to 1.12 (95% CI, -3.28 to 5.53) for moxifloxacin. All-cause mortality showed a similarly imbalanced pattern: only one death (0.78%) occurred in the ertapenem group, compared with 7.41% in the meropenem group, 2.40% in the cefoperazone–sulbactam group, and 2.73% in the moxifloxacin group. These crude differences translated into higher mortality risks for all three comparators relative to ertapenem, with RDs of -6.63% (95% CI, -11.30 to -1.96), -1.62% (95% CI, -4.71 to 1.46), and -1.95% (95% CI, -4.58 to 0.68), respectively. Infection-related and intra-abdominal infection–related deaths exhibited the same pattern, with no events in the ertapenem cohort versus 2.96%, 1.60%, and 1.82% in the meropenem, cefoperazone–sulbactam, and moxifloxacin groups, yielding RDs of -2.96% (95% CI, -5.82 to -0.10), -1.60% (95% CI, -3.80 to 0.60), and -1.82% (95% CI, -3.58 to -0.05). Secondary outcomes also deviated across treatment arms. Median duration of antibiotic therapy ranged from 138.5 hours (IQR, 106.4 to 185.7) in the moxifloxacin group to 156.5 hours (IQR, 107.6 to 209.0) in the meropenem group, with mean differences (MDs) versus ertapenem of -11.46 hours (95% CI, -28.02 to 5.10), -6.47 hours (95% CI, -22.15 to 9.22), and -0.47 hours (95% CI, -14.55 to 13.61) for meropenem, cefoperazone–sulbactam, and moxifloxacin, respectively. Median length of hospitalization was 10.0 days in both the ertapenem and moxifloxacin groups, 12.0 days in the meropenem group, and 11.0 days in the cefoperazone–sulbactam group, corresponding to crude MDs versus ertapenem of -2.69 days (95% CI, -4.74 to -0.65), -3.13 days (95% CI, -5.54 to -0.72), and -0.01 days (95% CI, -1.64 to 1.61) for meropenem, cefoperazone–sulbactam, and moxifloxacin. Collectively, these unadjusted findings highlight substantial confounding and baseline imbalance, supporting the necessity of IPTW to enable more reliable comparisons across treatment regimens.

**Table 3:** Primary and secondary outcomes across the four treatment groups before IPTW adjustment.

Outcomes	Ertapenem (n = 129)	Meropenem (n = 135)	Cefoperazone-Sulbactam (n = 125)	Moxifloxacin (n = 220)
Cured/Improved (%), RD (95% CI)	124 (96.12) Reference	117 (86.67) RD, 9.46 (2.83 to 16.09)	113 (90.40) RD, 5.72 (-0.42 to 11.87)	209 (95.00) RD, 1.12 (-3.28 to 5.53)
All-cause Death (%), RD (95% CI)	1 (0.78) Reference	10 (7.41) RD, -6.63 (-11.30 to -1.96)	3 (2.40) RD, -1.62 (-4.71 to 1.46)	6 (2.73) RD, -1.95 (-4.58 to 0.68)
Infection-related Death (%), RD (95% CI)	0 (0.00) Reference	4 (2.96) RD, -2.96 (-5.82 to -0.10)	2 (1.60) RD, -1.60 (-3.80 to 0.60)	4 (1.82) RD, -1.82 (-3.58, -0.05)
Intra-abdominal Infection-related Death (%), RD (95% CI)	0 (0.00) Reference	4 (2.96) RD, -2.96 (-5.82 to -0.10)	2 (1.60) RD, -1.60 (-3.80 to 0.60)	3 (1.36) RD, -1.36 (-2.90 to 0.17)
Duration of antibiotic treatment [hours (median, IQR)], MD (95% CI)	141.7 (108.9, 183.8) Reference	156.5 (107.6, 209.0) MD, -11.46 (-28.02 to 5.10)	154.2 (110.6, 193.1) MD, -6.47 (-22.15 to 9.22)	138.5 (106.4, 185.7) MD, -0.47 (-14.55 to 13.61)
Length of Hospitalization [days (median, IQR)], MD (95% CI)	10.0 (7.0, 14.0) Reference	12.0 (8.0, 18.0) MD, -2.69 (-4.74 to -0.65)	11.0 (8.0, 16.0) MD, -3.13 (-5.54 to -0.72)	10.0 (7.0, 13.25) MD, -0.01 (-1.64 to 1.61)

RD, risk difference; CI, confidence interval; IQR, interquartile range; MD, mean difference.

### 3.4 IPTW-adjusted primary and secondary outcomes

After applying inverse probability of treatment weighting to balance baseline characteristics across the four treatment groups, the comparative effectiveness estimates became substantially more comparable (**Table 4**). In the IPTW-adjusted analysis, clinical cure or improvement rates showed no statistically significant differences between ertapenem and the three comparator regimens. Relative to ertapenem, the adjusted RDs were 6.44% (95% CI, -0.42 to 13.30) for meropenem, 5.14% (95% CI, -1.27 to 11.55) for cefoperazone–sulbactam, and 1.80% (95% CI, -3.33 to 6.92) for moxifloxacin.

**Table 4:** IPTW-adjusted risk differences and mean differences for primary and secondary outcomes across the four treatment groups.

Outcomes	Ertapenem	Meropenem	Cefoperazone-Sulbactam	Moxifloxacin
Cured/Improved (%), RD (95% CI)	Reference	6.44 (-0.42 to 13.30)	5.14 (-1.27 to 11.55)	1.80 (-3.33 to 6.92)
All-cause Death (%), RD (95% CI)	Reference	-4.55 (-9.49 to 0.39)	-0.77 (-4.18 to 2.63)	-1.51 (-4.84 to 1.81)
Infection-related Death (%), RD (95% CI)	Reference	-2.60 (-5.59 to 0.38)	-1.46 (-3.61 to 0.70)	-1.76 (-3.72 to 0.20)
Intra-abdominal Infection-related Death (%), RD (95% CI)	Reference	-2.60 (-5.59 to 0.38)	-1.46 (-3.61 to 0.70)	-1.32 (-3.02 to 0.38)
Duration of antibiotic treatment, hours, MD (95% CI)	Reference	-21.47 (-39.65 to -3.29)	-13.30 (-29.33 to 2.74)	-9.12 (-24.22 to 5.98)
Length of Hospitalization, days, MD (95% CI)	Reference	-3.09 (-5.28 to -0.91)	-3.38 (-5.86 to -0.90)	-0.87 (-2.73 to 0.99)

RD, risk difference; CI, confidence interval; MD, mean difference.

**Figure 3:**
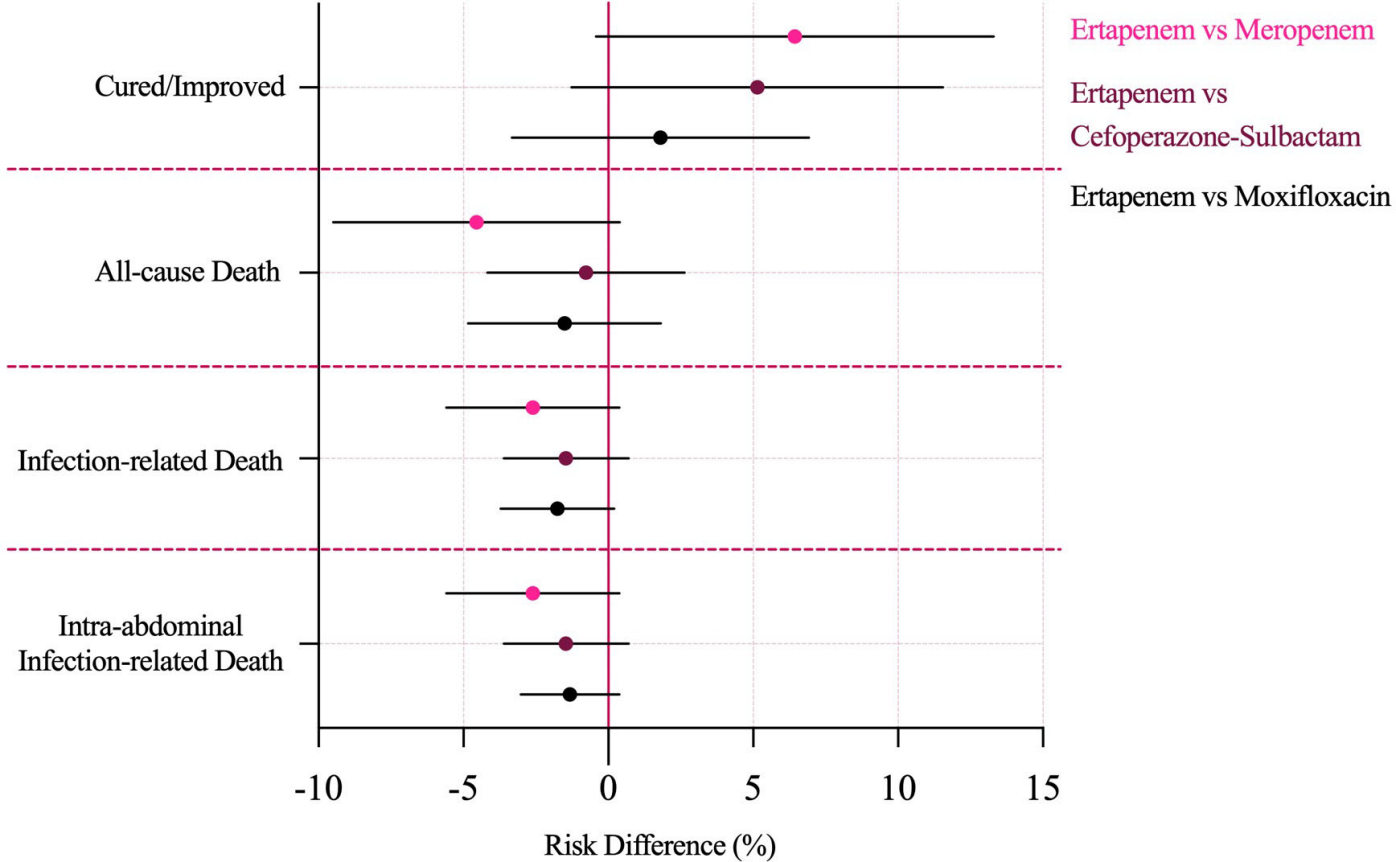
IPTW-adjusted risk differences for clinical outcomes. IPTW, inverse probability of treatment weighting.

Mortality outcomes similarly showed no significant adjusted differences. For all-cause mortality, the IPTW-adjusted RDs versus ertapenem were -4.55% (95% CI, -9.49 to 0.39) for meropenem, -0.77% (95% CI, -4.18 to 2.63) for cefoperazone–sulbactam, and -1.51% (95% CI, -4.84 to 1.81) for moxifloxacin. Infection-related mortality and intra-abdominal infection–related mortality demonstrated consistent patterns: adjusted RDs ranged from -2.60% (95% CI, -5.59 to 0.38) for meropenem to -1.32% (95% CI, -3.02 to 0.38) for moxifloxacin, with all confidence intervals spanning zero. The corresponding IPTW-adjusted forest plot of risk differences illustrates the absence of meaningful differences between ertapenem and comparator therapies (**Figure 3**, overall RD forest plot).

**Figure 4:**
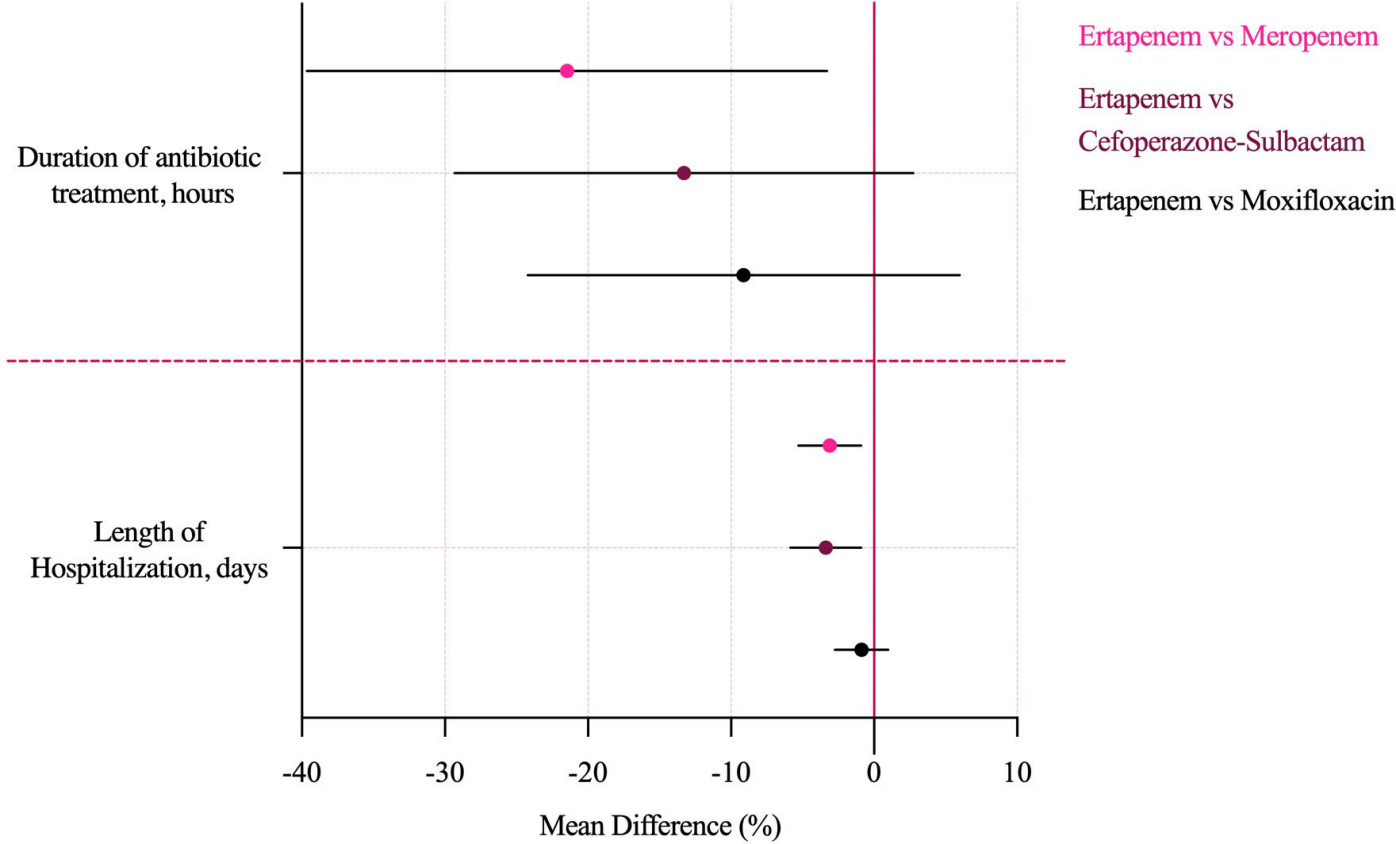
IPTW-adjusted mean differences for secondary outcomes. IPTW, inverse probability of treatment weightings.

For secondary outcomes, IPTW also attenuated crude differences. Duration of antibiotic therapy was shorter in all three comparator groups relative to ertapenem, and this difference reached statistical significance for meropenem (MD -21.47 hours, 95% CI -39.65 to -3.29), while the reductions with cefoperazone–sulbactam and moxifloxacin were not statistically significant. Length of hospitalization was significantly shorter with meropenem and cefoperazone–sulbactam (MD -3.09 days, 95% CI -5.28 to -0.91; and -3.38 days, 95% CI -5.86 to -0.90, respectively), whereas moxifloxacin showed no significant difference compared with ertapenem (**Figure 4**, overall MD forest plot).

### 3.5 Age-stratified subgroup analyses

The raw unweighted sample sizes and corresponding IPTW effective sample sizes for each age subgroup are summarized in **Supplementary Table 1**.

Age-stratified IPTW analyses were conducted for patients aged 65–70, 71–80, and ≥ 81 years, with ertapenem serving as the reference regimen. In the subgroup aged 65–70 years, ertapenem demonstrated higher cure or improvement rates than all three comparator antibiotics (**Table 5**). The adjusted RD for clinical cure was 10.25% (95% CI 2.10 to 18.39) compared with meropenem, 4.56% (95% CI -0.60 to 9.71) compared with cefoperazone–sulbactam, and 1.07% (95% CI -1.02 to 3.16) compared with moxifloxacin. Consistent directional differences were observed for mortality outcomes. For all-cause death, ertapenem showed lower event rates, with RDs of -8.24% (95% CI -15.47 to -1.02), -1.32% (95% CI -3.89 to 1.25), and -1.07% (95% CI -3.16 to 1.02) relative to meropenem, cefoperazone–sulbactam, and moxifloxacin, respectively. Infection-related and intra-abdominal mortality demonstrated similar patterns, with ertapenem consistently exhibiting lower crude mortality. Differences in secondary outcomes were modest; comparator regimens tended to have shorter antibiotic durations, while length of hospitalization differed only slightly between groups.

**Table 5:** IPTW-adjusted clinical and safety outcomes in patients aged 65–70 years treated with ertapenem versus comparator regimens.

Outcomes	Ertapenem	Meropenem	Cefoperazone-Sulbactam	Moxifloxacin
65 ≤ Age ≤ 70				
Cured/Improved (%), RD (95% CI)	Reference	10.25 (2.10, 18.39)	4.56 (-0.60, 9.71)	1.07 (-1.02, 3.16)
All-cause Death (%), RD (95% CI)	Reference	-8.24 (-15.47, -1.02)	-1.32 (-3.89, 1.25)	-1.07 (-3.16, 1.02)
Infection-related Death (%), RD (95% CI)	Reference	-3.77 (-8.95, 1.41)	-1.32 (-3.89, 1.25)	-1.07 (-3.16, 1.02)
Intra-abdominal Infection-related Death (%), RD (95% CI)	Reference	-3.77 (-8.95, 1.41)	-1.32 (-3.89, 1.25)	0.00 (0.00, 0.00)
Duration of antibiotic treatment, hours, MD (95% CI)	Reference	-24.21 (-54.80, 6.39)	-11.98 (-35.65, 11.69)	-5.52 (-27.63, 16.59)
Length of Hospitalization, days, MD (95% CI)	Reference	-2.56 (-5.91, 0.79)	-1.60 (-5.20, 1.99)	1.22 (-1.31, 3.75)

RD, risk difference; CI, confidence interval; MD, mean difference.

Among patients aged 71–80 years, between-group differences were smaller and largely centered around the null (**Table 6**). Adjusted RDs for cure or improvement were -1.46 (95% CI -10.57 to 7.65) for meropenem, 4.13 (95% CI -7.55 to 15.80) for cefoperazone–sulbactam, and 0.37 (95% CI -8.90 to 9.64) for moxifloxacin. Mortality outcomes likewise showed small differences, with RDs close to zero and wide confidence intervals. Mean differences in antibiotic duration were -8.50 hours, -2.52 hours, and 3.85 hours for meropenem, cefoperazone–sulbactam, and moxifloxacin, respectively, compared with ertapenem, and length of hospitalization was slightly longer with meropenem and cefoperazone–sulbactam.

**Table 6:** IPTW-adjusted clinical and safety outcomes in patients aged 71–80 years treated with ertapenem versus comparator regimens.

Outcomes	Ertapenem	Meropenem	Cefoperazone-Sulbactam	Moxifloxacin
71 ≤ Age ≤ 80				
Cured/Improved (%), RD (95% CI)	Reference	-1.46 (-10.57, 7.65)	4.13 (-7.55, 15.80)	0.37 (-8.90, 9.64)
All-cause Death (%), RD (95% CI)	Reference	0.82 (-5.45, 7.08)	1.54 (-5.04, 8.12)	-1.33 (-8.40, 5.74)
Infection-related Death (%), RD (95% CI)	Reference	-1.42 (-4.69, 1.84)	0.00 (0.00, 0.00)	-3.33 (-8.30, 1.63)
Intra-abdominal Infection-related Death (%), RD (95% CI)	Reference	-1.42 (-4.69, 1.84)	0.00 (0.00, 0.00)	-3.33 (-8.30, 1.63)
Duration of antibiotic treatment, hours, MD (95% CI)	Reference	-8.50 (-33.32, 16.31)	-2.52 (-27.06, 22.03)	3.85 (-18.14, 25.84)
Length of Hospitalization, days, MD (95% CI)	Reference	-3.74 (-7.02, -0.45)	-4.97 (-8.75, -1.18)	-1.81 (-5.07, 1.45)

RD, risk difference; CI, confidence interval; MD, mean difference.

In the subgroup aged ≥81 years, numerical differences were larger, although all confidence intervals continued to cross zero (**Table 7**). Cure or improvement RDs comparing ertapenem with meropenem, cefoperazone–sulbactam, and moxifloxacin were 19.55 (95% CI -1.04 to 40.15), 8.80 (95% CI -10.25 to 27.75), and 6.15 (95% CI -11.50 to 23.81), respectively. For all-cause mortality, the corresponding RDs were -10.91%, -4.11%, and -2.74%, and similar patterns were seen for infection-related and intra-abdominal infection–related death. Secondary outcomes showed longer antibiotic durations for all comparator regimens, particularly meropenem and cefoperazone–sulbactam, while reductions in hospitalization length were modest and varied among treatment groups.

**Table 7:** IPTW-adjusted clinical and safety outcomes in patients aged ≥81 years treated with ertapenem versus comparator regimens.

Outcomes	Ertapenem	Meropenem	Cefoperazone-Sulbactam	Moxifloxacin
81 ≤ Age				
Cured/Improved (%), RD (95% CI)	Reference	19.55 (-1.04, 40.15)	8.80 (-10.15, 27.75)	6.15 (-11.50, 23.81)
All-cause Death (%), RD (95% CI)	Reference	--10.91 (-22.92, 1.10)	-4.11 (-11.98, 3.77)	-2.74 (-8.08, 2.59)
Infection-related Death (%), RD (95% CI)	Reference	-3.69 (-10.82, 3.45)	-4.11 (-11.98, 3.77)	0.00 (0.00, 0.00)
Intra-abdominal Infection-related Death (%), RD (95% CI)	Reference	-3.69 (-10.82, 3.45)	-4.11 (-11.98, 3.77)	0.00 (0.00, 0.00)
Duration of antibiotic treatment, hours, MD (95% CI)	Reference	-47.06 (-91.96, -2.16)	-36.82 (-70.96, -2.67)	-43.26 (-79.09, -7.43)
Length of Hospitalization, days, MD (95% CI)	Reference	-2.29 (-5.24, 0.66)	-4.44 (-10.71, 1.84)	-3.42 (-7.68, 0.83)

RD, risk difference; CI, confidence interval; MD, mean difference.

In age-stratified IPTW analyses, treatment effects varied modestly across geriatric subgroups. Among patients aged 65–70 years, ertapenem demonstrated the most favorable outcomes, with a significantly higher clinical cure/improvement rate compared with meropenem (RD 10.25, 95% CI 2.10 to 18.39) and consistently lower all-cause and infection-related mortality relative to all three comparator antibiotics. In the 71–80-year subgroup, between-group differences were smaller and largely centered around the null; meropenem showed a slightly higher cure rate than ertapenem, but confidence intervals crossed zero, and mortality and secondary outcomes remained comparable across regimens. In patients aged ≥81 years, ertapenem again showed numerically higher cure rates and lower mortality than comparators, though all confidence intervals included zero, reflecting reduced precision in this oldest group. Overall, aside from a significant advantage for ertapenem in the 65–70-year subgroup, no consistent age-related effect modification was observed, and treatment outcomes were broadly similar across regimens in older patients.

### 3.6 Subgroup analyses by infection source

Raw unweighted sample sizes and IPTW effective sample sizes by source of cIAI and treatment group are provided in **Supplementary Table 2**.

IPTW-adjusted subgroup analyses were performed to evaluate whether infection source (gastrointestinal *vs*. non-gastrointestinal) modified the comparative effectiveness of ertapenem relative to the three comparator regimens.

Among patients with gastrointestinal cIAI, treatment outcomes were broadly comparable across all antibiotics (**Table 8**). Clinical cure or improvement rates showed no statistically significant differences between ertapenem and any comparator, with adjusted RDs of 4.34 (95% CI -2.64 to 11.32) for meropenem, -0.25 (95% CI -9.74 to 9.23) for cefoperazone–sulbactam, and 0.65% (95% CI -5.15 to 6.46) for moxifloxacin. Mortality outcomes were directionally favorable for ertapenem compared with meropenem, including lower all-cause mortality and significantly reduced infection-related mortality (RD -3.14%, 95% CI -6.22 to -0.06). Differences for cefoperazone–sulbactam and moxifloxacin were smaller but remained consistent in direction. Secondary outcomes also favored ertapenem, with shorter antibiotic durations for all three comparator therapies and significantly reduced hospitalization length.

**Table 8:** IPTW-adjusted clinical and secondary outcomes in patients with gastrointestinal source cIAI treated with ertapenem versus comparator regimens.

Outcomes	Ertapenem	Meropenem	Cefoperazone-Sulbactam	Moxifloxacin
Gastrointestinal				
Cured/Improved (%), RD (95% CI)	Reference	4.34 (-2.64, 11.32)	-0.25 (-9.74, 9.23)	0.65 (-5.15, 6.46)
All-cause Death (%), RD (95% CI)	Reference	-3.80 (-8.96, 1.36)	-5.00 (-12.65, 2.65)	-1.80 (-6.07, 2.47)
Infection-related Death (%), RD (95% CI)	Reference	-3.14 (-6.22, -0.06)	-3.06 (-7.61, 1.49)	-2.12 (-4.24, 0.00)
Intra-abdominal Infection-related Death (%), RD (95% CI)	Reference	-3.14 (-6.22, -0.06)	-3.06 (-7.61, 1.49)	-1.59 (-3.45, 0.27)
Duration of antibiotic treatment, hours, MD (95% CI)	Reference	-22.13 (-43.18, -1.08)	-18.63 (-45.18, 7.92)	-15.59 (-31.31, 0.14)
Length of Hospitalization, days, MD (95% CI)	Reference	-2.84 (-5.18, -0.50)	-1.95 (-4.79, 0.89)	-1.18 (-3.27, 0.91)

RD, risk difference; CI, confidence interval; MD, mean difference.

In contrast, among patients with non-gastrointestinal cIAI, ertapenem demonstrated clearer advantages over comparator treatments (**Table 9**). Clinical cure or improvement was significantly higher with ertapenem, with large positive RDs observed for meropenem (16.39, 95% CI 4.02 to 28.76), cefoperazone–sulbactam (29.87, 95% CI 10.11 to 49.63), and moxifloxacin (7.19, 95% CI -6.68 to 21.05). Mortality outcomes also favored ertapenem: both all-cause and infection-related deaths were lower compared with meropenem and cefoperazone–sulbactam, although confidence intervals occasionally reached zero. Differences in treatment duration were more variable, with longer antibiotic courses observed for cefoperazone–sulbactam and moxifloxacin, whereas hospitalization tended to be shorter with ertapenem, especially relative to meropenem (MD -4.33 days, 95% CI -9.19 to 0.53).

**Table 9:** IPTW-adjusted clinical and secondary outcomes in patients with non-gastrointestinal source cIAI treated with ertapenem versus comparator regimens.

Outcomes	Ertapenem	Meropenem	Cefoperazone-Sulbactam	Moxifloxacin
Non-Gastrointestinal				
Cured/Improved (%), RD (95% CI)	Reference	16.39 (4.02, 28.76)	29.87 (10.11, 49.63)	7.19 (-6.68, 21.05)
All-cause Death (%), RD (95% CI)	Reference	-8.11 (-17.13, 0.90)	-8.16 (-19.50, 3.17)	-6.57 (-15.45, 2.32)
Infection-related Death (%), RD (95% CI)	Reference	0.00 (0.00, 0.00)	-8.16 (-19.50, 3.17)	-6.57 (-15.45, 2.32)
Intra-abdominal Infection-related Death (%), RD (95% CI)	Reference	0.00 (0.00, 0.00)	-8.16 (-19.50, 3.17)	-6.57 (-15.45, 2.32)
Duration of antibiotic treatment, hours, MD (95% CI)	Reference	-18.99 (-50.98, 13.00)	25.32 (-7.29, 57.93)	-7.25 (-32.99, 18.49)
Length of Hospitalization, days, MD (95% CI)	Reference	-4.33 (-9.19, 0.53)	-3.94 (-10.97, 3.09)	-3.59 (-9.01, 1.82)

RD, risk difference; CI, confidence interval; MD, mean difference.

Overall, these findings suggest that ertapenem performs comparably to broad-spectrum alternatives in gastrointestinal cIAI, but may offer a distinct clinical advantage in non-gastrointestinal cIAI, demonstrating higher cure rates and consistently lower mortality relative to the comparator regimens.

## 4 Discussion

cIAI in older adults remains a major clinical challenge because of atypical presentations, multimorbidity, and age-related immune dysfunction. International guidelines consistently emphasize that timely source control and appropriate empirical antimicrobial therapy are critical determinants of outcome in these infections (Solomkin et al., 2010; Mazuski et al., 2017; Sartelli et al., 2017a, 2021). In this IPTW-adjusted real-world study, ertapenem demonstrated clinical effectiveness comparable to meropenem, cefoperazone–sulbactam, and moxifloxacin for empirical management of cIAI in elderly patients. These findings are broadly consistent with guideline recommendations that encourage tailoring antimicrobial spectrum to individual risk profiles while avoiding unnecessarily broad coverage (Solomkin et al., 2010; Mazuski et al., 2017; Sartelli et al., 2017a).

Our results align with randomized controlled trials and meta-analyses showing that once-daily ertapenem is non-inferior to piperacillin–tazobactam or ceftriaxone-based combinations for community-acquired cIAI and other complicated infections. In these studies, ertapenem achieved similar clinical cure rates and safety profiles compared with broader-spectrum comparators (Solomkin et al., 2003; Namias et al., 2007; Falagas et al., 2008; An et al., 2009). Its pharmacokinetic and pharmacodynamic properties, including predictable exposure and convenient once-daily dosing, further support its use in older adults who often face complex care pathways and polypharmacy (Nix et al., 2004).

From an antimicrobial stewardship perspective, ertapenem offers several potential advantages. Unlike meropenem, ertapenem lacks antipseudomonal activity and has a narrower spectrum that may reduce collateral selection pressure for carbapenem-resistant Enterobacterales and multidrug-resistant non-fermenters (Solomkin et al., 2010; Mazuski et al., 2017; Sartelli et al., 2017a, 2021). Older adults, particularly those with frequent healthcare exposure and immunosenescence, are at increased risk of colonization and infection with multidrug-resistant organisms (Crooke et al., 2019). In such settings, reserving broader-spectrum carbapenems for patients with clear risk factors for resistant pathogens while using ertapenem for lower-risk cIAI may help balance individual patient outcomes with ecological considerations.

Subgroup analyses in our cohort suggested that patients aged 65–70 years and those with non-gastrointestinal infection sources may experience more favorable outcomes with ertapenem. Although these trends are clinically plausible, non-gastrointestinal infections may involve pathogens well covered by ertapenem, and younger elderly patients may have greater physiological reserve; they must be interpreted with caution. Several subgroups had a limited effective sample size after weighting, and confidence intervals were wide. Moreover, confounding by indication remains possible, as clinicians may preferentially prescribe meropenem for patients perceived as more severely ill, even after IPTW adjustment (Hernan and Robins, 2020). These subgroup results should therefore be regarded as exploratory and hypothesis-generating rather than definitive. In particular, any residual confounding related to unmeasured illness severity or frailty would be expected to favor ertapenem over broader-spectrum comparators such as meropenem.

The absence of comprehensive microbiological data in this study limits our ability to explore organism-specific treatment responses or resistance patterns. This is particularly relevant for ertapenem, which is inactive against Pseudomonas aeruginosa; our findings are most directly applicable to empirical therapy in settings where Pseudomonas risk is low or can be reliably stratified (Solomkin et al., 2010; Mazuski et al., 2017; Sartelli et al., 2017a, 2021). In addition, safety outcomes were not systematically captured in our dataset. While previous studies have suggested a generally favorable tolerability profile for ertapenem and have raised concerns about coagulation disorders with cefoperazone-containing regimens (Falagas et al., 2008; Miao et al., 2023), our study cannot provide comparative safety conclusions and any safety-related discussion should be viewed as contextual rather than data-driven.

Despite these limitations, the present study adds pragmatic real-world evidence to the existing trial and guideline literature by focusing specifically on elderly patients with cIAI, a population characterized by immunosenescence and high vulnerability to adverse outcomes (Crooke et al., 2019). Overall, our findings suggest that ertapenem may be an appropriate empirical option for selected older adults with cIAI. Prospective, ideally multicenter, studies incorporating detailed microbiological data, severity-of-illness scores, frailty measures, and systematically collected safety endpoints are needed to validate and refine individualized, stewardship-conscious treatment strategies for this high-risk group.

## 5 Limitations and future directions

This study has several important limitations. First, as with all observational research, residual confounding cannot be fully excluded despite the robust application of inverse probability of treatment weighting. Although IPTW achieved an excellent balance across measured covariates, unmeasured factors, particularly clinical severity indicators such as organ dysfunction scores, septic shock status, frailty, and physicians’ subjective assessment, may have influenced empirical antibiotic selection. In clinical practice, meropenem is often chosen for patients perceived as more critically ill, raising the possibility of confounding by indication that could bias subgroup estimates. Such confounding by indication would most plausibly bias estimates in favor of ertapenem and against meropenem, given the preferential use of broader-spectrum agents in patients perceived as more severely ill or frail, and should be acknowledged when interpreting subgroup findings.

Second, microbiological data, including pathogen distribution, culture positivity rates, and antimicrobial resistance profiles, were not available in a standardized form for the full cohort. As a result, we could not evaluate whether differences in treatment response were related to underlying microbial etiology, the presence of resistant organisms, or pathogen-specific virulence. This limitation is particularly relevant for ertapenem, which lacks antipseudomonal activity and constrains our ability to explore mechanisms underlying clinical outcomes. The absence of comprehensive microbiology data also limits the relevance of our findings to early empirical treatment rather than definitive pathogen-directed therapy.

Third, although predefined subgroup analyses were conducted, some subgroups—especially patients aged ≥81 years and those with non-gastrointestinal infection sources—had reduced effective sample sizes after IPTW. Consequently, subgroup estimates should be interpreted as exploratory and hypothesis-generating rather than confirmatory. Wide confidence intervals in some strata further indicate uncertainty around these estimates.

Fourth, safety outcomes were not evaluated in this study. Although ertapenem generally has favorable tolerability in the literature, our dataset did not include consistent or validated adverse event reporting. Therefore, no conclusions regarding the comparative safety of the four regimens can be drawn from these results, and any discussion of safety should be considered contextual rather than based on the study findings.

Finally, this was a single-center study conducted in a tertiary academic hospital in northeast China, which may limit generalizability to other healthcare settings, antimicrobial resistance patterns, and patient populations with differing comorbidities or immunosuppressive conditions. Prospective multicenter studies that incorporate microbiological data, illness severity measures, and safety outcomes are needed to validate and extend these findings.

Future research should incorporate granular microbiological and resistance data to better characterize pathogen-specific responses and to understand how ertapenem performs in infections caused by resistant organisms, including ESBL-producing Enterobacterales and Pseudomonas risk scenarios. Prospective cohort studies or pragmatic randomized trials integrating severity-of-illness scores, immunological biomarkers, and frailty assessments would help address residual confounding. In addition, studies evaluating safety outcomes, ecological impact, and long-term functional recovery in elderly cIAI patients are needed. Finally, investigations that focus on immunosenescent or functionally immunocompromised elderly populations may provide deeper insights into host–pathogen interactions and optimal empirical antibiotic strategies in this vulnerable group.

## 6 Conclusion

In this IPTW-adjusted real-world analysis of elderly adults with complicated intra-abdominal infection, ertapenem demonstrated clinical effectiveness comparable to meropenem, cefoperazone–sulbactam, and moxifloxacin as empirical monotherapy. These findings indicate that ertapenem may serve as a reasonable empirical option for selected older patients with cIAI in routine clinical practice. Subgroup observations in younger elderly patients and in non-gastrointestinal infection sources should be considered exploratory, given the limited effective sample size and potential residual confounding.

In the context of rising antimicrobial resistance and the increased vulnerability associated with immunosenescence, optimizing empirical antibiotic choices while avoiding unnecessary broad-spectrum exposure remains essential. Future prospective and multicenter studies incorporating microbiological data, severity assessments, frailty measures, and safety outcomes are needed to confirm these results and refine individualized treatment strategies for this high-risk population.

## Data Availability

The raw data supporting the conclusions of this article are available from the corresponding author upon reasonable request. Requests to access these datasets should be directed to YS, sunyao0507@jlu.edu.cn.
