# Supplementary material for: Comparative effectiveness of ertapenem versus other empirical antibiotics in elderly patients with complicated intra-abdominal infection: a real-world inverse probability of treatment weighting study
Source: Front Cell Infect Microbiol. 2026 Jan 16;15:1759636. doi: 10.3389/fcimb.2025.1759636 (PMC12855448; doi:10.3389/fcimb.2025.1759636)
Supplement: Supplementary file 1 [file Table1.docx]

Supplementary Material

**Supplementary Table 1.** Raw and IPTW Effective Sample Sizes for Age-Stratified Subgroup Analyses

| Age Groups, years | Ertapenem | | Meropenem | | Cefoperazone-Sulbactam | | Moxifloxacin | | Overall | |
| --- | --- | --- | --- | --- | --- | --- | --- | --- | --- | --- |
|  | N | ESS | N | ESS | N | ESS | N | ESS | N | ESS |
| 65–70 | 50 | 40.77 | 46 | 35.17 | 54 | 50.66 | 102 | 87.93 | 252 | 211.50 |
| 71–80 | 54 | 45.50 | 65 | 54.58 | 44 | 42.09 | 85 | 66.03 | 248 | 206.56 |
| ≥ 81 | 25 | 21.67 | 24 | 20.52 | 27 | 25.69 | 33 | 25.12 | 109 | 88.64 |

IPTW: inverse probability of treatment weighting; N: unweighted raw sample size; ESS: effective sample size.

**Supplementary Table 2.** Raw and IPTW Effective Sample Sizes by Source of cIAI and Treatment Group

| Source of cIAI | Ertapenem | | Meropenem | | Cefoperazone-Sulbactam | | Moxifloxacin | | Overall | |
| --- | --- | --- | --- | --- | --- | --- | --- | --- | --- | --- |
|  | N | ESS | N | ESS | N | ESS | N | ESS | N | ESS |
| Gastrointestinal | 99 | 82.95 | 101 | 83.44 | 101 | 96.57 | 203 | 183.78 | 504 | 439.83 |
| Non-gastrointestinal | 30 | 26.98 | 34 | 31.69 | 24 | 21.86 | 17 | 14.04 | 105 | 70.99 |

IPTW: inverse probability of treatment weighting; cIAI: complicated intra-abdominal infection; N: unweighted raw sample size; ESS: effective sample size.
